# Secure Spectrum Access, Routing, and Hybrid Beamforming in an Edge-Enabled mmWave Massive MIMO CRN-Based Internet of Connected Vehicle (IoCV) Environments

**DOI:** 10.3390/s22155647

**Published:** 2022-07-28

**Authors:** Deepanramkumar Pari, Jaisankar Natarajan

**Affiliations:** School of Computer Science and Engineering, Vellore Institute of Technology, Vellore 632014, India; deepanramkumar.p2019@vitstudent.ac.in

**Keywords:** cognitive radio network (CRN), 6G, Internet of connected vehicles (IoCV), spectrum sensing and handoff, hybrid beamforming

## Abstract

A cognitive radio network (CRN) is integrated with the Internet of Connected Vehicles (IoCV) in order to address spectrum scarcity and communication reliability issues. However, it is limited, possessing less throughput, a low packet delivery ratio, high latency, and high mobility in the spectrum. In this research study, the existing issues are addressed by proposing a 6G cognitive radio network–Internet of connected vehicles (6GCRN–IoCV) approach. Initially, all the entities such as secondary users (SUs), primary users (PUs), and pedestrians are authenticated in blockchain to ensure security. The edge-assisted roadside units (ERSU) initiate clustering only for authenticated SUs using the improved DBSCAN algorithm in consideration of several metrics. The ERSU then generates an intersection-aware map using the spatial and temporal-based logistic regression algorithm (STLR) to reduce collisions in the intersection. The spectrum utilization is improved by performing spectrum sensing in which all the SUs involved in spectrum sensing use lightweight convolutional neural networks (Lite-CNN) in consideration of several metrics and provide the sensing report to the fusion center (FC) in an encrypted manner to reduce the spectrum scarcity and security issues. The communications between the SUs are necessary to avoid risks in the IoCV environment. Hence, optimal routing is performed using the Dingo Optimization Algorithm (DOA), which increases throughput and packet delivery ratio. Finally, communication reliability is enhanced by performing hybrid beamforming, and this exploits the multi-agent-based categorical Deep-Q Network (categorical DQN), which increases spectral efficiency based on its adaptive intelligent behavior. The proposed study is simulated using the SUMO and OMNeT++ simulation tools and the performances are validated with existing works using several performance metrics. The result of the simulation shows that the proposed work performs better than the existing approaches.

## 1. Introduction

A cognitive radio network (CRN) is an emerging technology for solving spectrum scarcity issues by sensing and utilizing unoccupied spectrums or spectrum holes, which also enhances spectral efficiency in the CRN [1]. Next-generation communication technologies such as 5G and 6G provide the connection for the Internet of Connected Vehicles (IoCV) (i.e., autonomous vehicles, self-driving cars) [2,3]. In the IoCV environment, efficient network management is performed by implementing the clustering of users [4]. In CRN, the spectrum utilization method allows unlicensed users or secondary users (SUs) to speculatively accomplish spectrum holes or cognitive white spaces that are not used by primary users (PUs) or licensed users. For spectrum sensing, many researchers have proposed machine learning (ML) and deep learning (DL) algorithms to enhance the capability of spectrum sensing [5,6,7,8]. However, the channel availability depends on the condition of PUs’ activity. The CRN network includes two main objectives: extreme reliable communication and effective spectrum utilization. However, spectrum sensing and access are divided into two classes: static and dynamic. In static spectrum sensing, some frequency bands are accessed by the SUs which means that they cannot access other spectrums in the channel. In the case of PU inactivity, static spectrum sensing leads to high transmission delay and spectrum inefficiency [9]. To overcome these issues, dynamic spectrum sensing and access are introduced into the CRN. In this case, the sensing spectrum is dynamically assigned to the SUs while PU inactivity leads to spectrum efficiency [10]. In dynamic spectrum sensing, the sensed spectrum is handed off by selecting a target channel, which is known as the mobility management of the spectrum [11,12]. Spectrum handoff includes three conditions:

- When the SU connection is failing due to mobility;
- When the sensed spectrum does not satisfy the requirements;
- When the inactivity of the PU is detected.

Therefore, the unoccupied spectrums are utilized by the SUs [13]. Several challenges occur during spectrum handoffs such as high latency and energy consumption. To overcome these issues, edge-based handoff (i.e., task offloading) is implemented for efficient data transmission [14]. However, routing is an important issue in CR-VANET. Network management is one of the major issues in the CR-VANET environment that was addressed by introducing adaptive clustering methods [15]. However, the clustering metrics are not enough, and frequent disconnections lead to unwanted energy consumption for SUs, thereby leading to high network traffic [16]. The traditional CR routing solutions are not suitable for CR-VANET due to the mobile nature of vehicles and the dynamic spectrum nature of the CRN. Hence, routing between vehicles is an important process for achieving a high packet delivery ratio and throughput [17,18]. In CRN, a massive multiple-input multiple-output (MIMO) system is introduced to handle high network traffic, in which MIMO considers only one key technology to increase the data rate in 5G or 6G communications [19]. The massive MIMO is implemented to overcome the general issues of MIMO such as low spectral efficiency and high energy consumption [20]. That single beamforming structure needs one radio frequency (RF) chain per antenna, which leads to high power consumption and costs. To resolve these issues, hybrid beamforming is introduced into the massive MIMO. Hybrid beamforming integrates high-dimensional analog and low-dimensional beamforming with a limited amount of RF chain [21]. Deep reinforcement learning (DRL) algorithms such as Q learning, Deep Q-Network (DQN), deep deterministic policy agent (DDPG), etc., are used for hybrid beamforming [22]. CR-VANET also integrates with cloud/edge/fog computing technologies to resolve resource utilization issues [23]. However, the issues with hybrid beamforming are listed below:

- Hardware efficiency;
- Computational efficiency;
- Spectral efficiency;
- Low SNR.

### 1.1. Motivation and Objectives

The main aim of this research is to manage the mobility of spectra and network traffic, which increases spectral efficiency for autonomous driving systems in CRN-IoCV environments. This research also addresses the problems of security, high mobility, high latency, and inefficient spectrum utilization in an edge-enabled 6G mmWave massive MIMO CRN-based Internet of Connected Vehicles (IoCV) environment. We are motivated by various problems addressed in the previous works, which are described as follows:

- **High Latency:** In existing works, all SUs send their information to the FC through the RSU; however, because RSUs have less storage, they do not have the capability to store their information, which leads to a high overload in RSUs that increases latency in the IoCV environment.
- **High Mobility in Spectrum Sensing:** Every secondary user (SU) is allowed for spectrum sensing in the primary user (PU). Due to the mobile nature of the SUs in the sensing environment, spectrum handoff is greatly affected, which leads to improper spectrum sensing and interference in the PUs.
- **Low Throughput:** For data transmission, the best routing path is required, but due to insecure routing, attackers might block the path which may cause congestion between the PUs and SUs in the IoCV environment, leading to low throughput.
- **High Hardware Complexity:** In hybrid beamforming, more phase shifters (PSs) are used for reducing energy consumption. By using more PSs, analysis is more difficult because of their hardware complexity, and this may lead to low spectral efficiency and cause a network delay. Using more RF chains in the precoder also increases high energy consumption and hardware complexity.

The main objective of this research is to manage the spectrum mobility and network traffic in edge-enabled 6G-CRN with low latency, energy efficiency, and spectrum efficiency. The other objectives of this research are listed as follows:

- To address the spectrum scarcity and mobility by performing dynamic spectrum sensing and handoff that increases the utilization of spectrums in the CR environment. SSDF and CPUF attacks are mitigated by encrypting the sensing reports.
- To address flexibility, spectral, and hardware efficiency by performing secure hybrid beamforming that generates dynamic beams by verifying CSI using blockchains that provide secure beams.
- To address the network traffic by performing secure routing, which increases high throughput and security during data transmission.
- To reduce energy consumption and latency by deploying edge-based RSU, which reduces latency during clustering and routing with the aid of past historical information.
- To ensure security by performing encryption for the sensing report which helps mitigate SSDF and PUF attacks during spectrum sensing and handoff.

### 1.2. Research Contributions

The secure and reliable 6GCRN-IoCV approach is introduced to address the problems faced by the existing methods such as less throughput, high spectrum mobility, and increased latency. The contributions of this study are as follows:

- The authenticity of all entities (PUs (ERSU), SUs, and pedestrians) in the network is ensured by blockchain. The authenticated nodes are clustered to manage the network by using the improved DBSCAN algorithm and exploiting several clustering metrics. The intersection-aware map is constructed by ERSU for reducing collision at an intersection by considering several metrics.
- The spectrum scarcity issue is addressed by the efficient utilization of spectrums by all SUs in the environment, which is performed by lightweight convolutional neural networks (Lite-CNN) exploding several metrics. All sensing reports by SUs are encrypted and provided to the fusion center (FC).
- The communication between the SUs is enhanced to achieve reliability in communication, which is carried out by optimal routing and employing the Dingo Optimization Algorithm (DOA) using routing metrics. The routing process increases throughput and enhances the packet’s delivery ratio.
- The spectral efficiency is improved by the execution of hybrid beamforming using the multi-agent-based categorical deep Q network (categorical DQN) in which agent actions are jointly assessed and hybrid beamforming is executed.

### 1.3. Paper Organization

**Figure 1:**
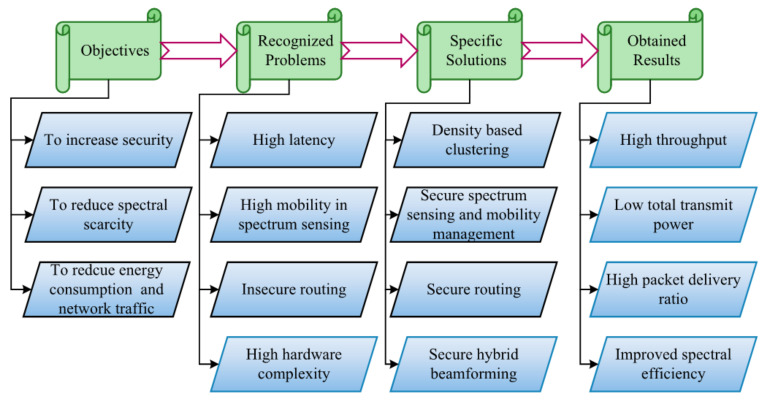
Design reasons for the 6GCRN-IoCV model.

The rest of the paper is organized as follows: Section 2 presents a survey of the existing works in which current gaps in the research are analyzed and addressed. Section 3 deals with specific problems, and research solutions are provided. Section 4 represents the detailed explanation of the proposed work with suitable pseudocodes and diagrams. Section 5 provides the experimental setup in which the simulation setup, comparative analysis, and research summary are clearly explained. Section 6 provides a detailed explanation of the conclusion of the proposed work. Table 1 represents the notations used in this work with corresponding descriptions, and Figure 1 illustrates the reasons for designing the 6GCRN-IoCV method.

## 2. Literature Survey

This section describes various previous studies that are correlated to sensing and accessing the spectrum securely with hybrid beamforming and handoff based on various techniques including ML and DL approaches. In addition, this section discusses the research gaps in these works, which are summarized below. The secure data processing framework in the edge-envisioned V2X environment using blockchain (BlockEd) was carried out in [24]. The proposed work uses a multilayer system model for BlockEd. The proposed work contains five layers for secure data processing such as the V2X layer where the primary users (RSU), secondary users (vehicles), and edge servers are present. In the task scheduling layer, secondary users request tasks, and they are provided with unique IDs, which are sent to the task administrator. The task administrator assigns a task and provides a unique task ID based on parameters such as task time and task type. Based on the information from the task administrator, the container management layer forms a container list that creates container maps. In the edge layer, the edge node controller requests the task from the container map and provides it accordingly to prevent any breakage. In the block management layer with information from the edge, the server is stored in a hash manner for high security. The authors of [25] ensured the security of IoV by introducing a blockchain-based batch authentication mechanism. The main aim of this research study is to perform authentication using blockchain and artificial intelligence methods in IoV. The proposed architecture includes five phases such as the initial setup, RSU registration and a message sign, authentication, and key management. At first, the vehicles and RSU are registered in the blockchain, and then it provides the signature to the registered vehicles and the RSU. Next, batch authentication is performed to reduce computational overhead in the environment. Finally, group keys are shared between the clusters to enhance security. The simulation result shows that the proposed work achieves better performance in terms of security, storage, and computation cost compared to existing works. Here, authentication is performed for both the vehicle and the RSU; however, this research study does not consider the PUF and the location for authentication, because PUF is a unique ID that varies from device to device. Therefore, it needs to be considered for authentication; otherwise, it reduces performance.

In [26], the malicious user was detected in the cognitive radio–Internet of Things (CR-IoT) environment for reliable cooperative radio spectrum sensing (CSS) using a machine learning (ML) algorithm. The ML algorithm used in this paper is the support vector machine algorithm (SVM). In the CR-IOT environment, both primary users (licensed users) and secondary users (unlicensed users) are present. Both are permitted in the local sensing of vacant spectrums using CSS, where the primary users have high priority. Regarding the vacant spectrum, a report is sent to the fusion center (FC), where the global decision is made. At FC, by using the SVM, algorithm users are classified as normal and malicious users. FC makes decisions based only on normal CR-IoT users. Here, the SVM algorithm is used for the classification of malicious and normal users; however, it requires a longer period of time for training, which leads to high latency. Hence, it is not suitable for large-scale environments. The authors of [27] proposed controlling MRCSC in multi-cell multi-channel multi-radio cognitive radio wireless networks using a cross-layer algorithm. By using MRCSC, both inter-flow and intra-flow interferences are considered. The proposed work consists of three stages: energy detection, matched filter detection, and cyclo-stationary detection. In the energy detection phase, the energy of nodes is detected according to the amount of transmission shared between the neighboring nodes. In matched filter detection, the number of malicious nodes is detected by using SNR. In cyclo-stationary detection, spectrum sensing takes place where the effective path is made by joining MRCSC using a cross-layer algorithm. Here, the cross-layer algorithm is used for joining MRCSC, which takes a lot of time to select an optimal path, leading to high latency. In addition, it does not consider the trust factor of the node, which leads to poor security.

The authors of [28] described a hierarchically assisted framework with hybrid dynamic spectrum access via a mobility-compatible database. The proposed work uses the Markov decision process (MDP) for spectrum access. In this proposed work, spectral maps are extracted from each node and stored in a database. The nodes are grouped as clusters, where the cluster head is assigned for accessing the spectral maps in a database for spectrum sensing. The cluster head selection is based on the ratio of successful connections in the database, the maximum number of spectral maps stored in that node, and the local mobility aggregation. For spectrum access information from the cluster, heads are taken for the MDP process where the spectral sensing effectively takes place. Here, the MDP process is used for spectrum sensing; therefore, extensive data are required for the MDP process. The authors of [29] proposed a method for efficient spectrum utilization through dynamic channel reservation and spectrum access. The proposed architecture includes three entities: the primary user (PU), the secondary user (SU), and the base station (BS). Here, a hybrid dynamic channel reservation algorithm is proposed for dynamically allocating the channels to the users. Dynamic channel allocation is used to enhance the dynamic spectrum’s access. Here, the available spectrum is classified into two classes: reserved and non-reserved bands. For spectrum access, the SUs are divided into two classes (high priority and low priority) based on the QoS, which increases the performance of dynamic spectrum access. The simulation result shows that the proposed work achieves better performance in terms of spectrum efficiency compared to existing works. Here, the SU accesses the spectrum based on its priority level, which is not enough for dynamic spectrum access, leading to spectrum scarcity. In addition, spectrum mobility is not concentrated, which leads to poor performance in spectrum access.

The authors of [30] proposed spectrum handoff using the DQN algorithm in a hybrid cognitive radio network. The proposed work includes two entities, a secondary base station (SBS) and a primary base station (PBS), in which the SBS predicts the channel availability for spectrum handoff. The PBS shares the spectrum using hybrid cognition that includes interweaving and underlaying. Here, both the PU and SU transmit by an adaptive modulation and coding method by considering channel state information (CSI) using active learning. Here, spectrum handoff conditions are satisfied to increase the throughput through the DQN algorithm which dynamically adjusts the SINR values. The simulation result demonstrates that the proposed work achieves better performance in terms of throughput, convergence rate, and handoff success rate. Here, CSI information is considered for spectrum handoff but it is not enough for spectrum handoff, which reduces the performance of the spectrum handoff. This work does not consider the trust factor of the SU, which leads to high data threats and poor security. The authors proposed a secure spectrum handoff method for cognitive radio networks which work against cognitive user emulation attacks in [31]. In this research, user identification is performed for every user during handoff to increase the level of security. Here, the handoff is performed based on the trust factor or value. The legitimacy of the user is evaluated by CCU by computing the trust value for mitigating the handoff threats. The new user is validated by PU and HCU. The experimental result shows that the proposed work achieves better performance in terms of throughput, transmission delay, false authentication, and detection rate. Here, the handoff is performed based on the trust value which is not enough for optimal spectrum handoff and needs to consider some important metrics such as channel availability, congestion, and SNR. Otherwise, it leads to poor handoff, that increases congestion and transmission delay. The authors of [32] proposed a spectrum handoff method for cognitive radio networks. The proposed work performs spectrum handoff by the emergence of PUs, which is predicted based on the channel utilization. The spectrum handoff reduces the collision between the SUs and PUs. Initially, the SU senses the available spectrum by considering channel utilization. For spectrum handoff, the proposed work includes two criteria: probability prediction and the inactive state. The SU selects the target channel for spectrum handoff, which prevents the collision in the network and reduces handoff delay. The simulation result shows that the proposed work achieves better performance in terms of collision probability, mean packet delay, capacity, handoff delay, and packet loss ratio compared to the existing work. Here, the target channel is predicted for the spectrum handoff; however, it provides poor results during the handoff due to the lack of channel characteristics. In addition, this research does not consider the security credentials for the SU during handoff, which leads to poor security.

In [33], V2X task offloading in a mobile edge computing (MEC)-assisted intelligent transportation system was performed in a costly manner. The proposed work uses integer linear programming (ILP), and the cost-effective heuristic algorithm for task offloading (CHAT) is used. In intelligent transportation systems (ITS), delays are the most salient problem. This proposed work uses an ILP algorithm that can form a normalized mathematical function for solving delay problems. For cost-effective and select servers at a minimum cost, the CHAT algorithm is used, which forms a task flow inset and selects one server from each set of free servers and assigns it in a hash manner. Here, the ILP algorithm is used for solving delay problems; however, it consumes more energy during task offloading, which reduces the performance of the work. Dynamically varying spectrum availability routing in cognitive radio networks (CRN) with full-duplex is used in [34]. The proposed work contains three stages: path discovery, channel selection, and path selection. In the path discovery stage, a set of feasible routes is determined by finding dynamic access to underutilized primary channels. During channel selection, the feasible paths are dynamically provided with stage channels. During path selection, using the full-duplex method, CRNs can be classified into in-band full-duplex (IBFD) CRNs and out-band full-duplex (OBFD) CRNs. For both IBFD and OBFD, the effective path time is calculated, indicating the idle path and busy path. Here, the full-duplex mode is used for path selection; however, it does not take the proper path metrics, which leads to inefficient path selection that reduces performance during spectrum access.

The authors proposed optimal routing based on social information for the CR-VANET environment, which increases the high packet delivery ratio and reduces the overhead ratio [35]. Here, the primary user’s social information is considered for evaluating spectrum holes. A social community partition algorithm is proposed for classifying the SUs into inter- and intra-community processes. For the inter-community process, this research proposes an optimized binary tree-based policy, and for the intra-community process, this paper utilizes a single-copy policy. The experimental result shows that the proposed work achieves better performance in terms of the packet delivery ratio and overhead ratio compared to existing work. Here, routing is performed based on the social character of the SU; however, it provides poor routing due to a lack of next forwarder characteristics and security, which leads to poor security and less throughput. Delay-tolerant routing for cognitive radio vehicular ad hoc networks was proposed in [36]. The proposed work architecture includes two components: delay-tolerant routing and a message scheduling method. At first, the relay node is selected based on the channel availability and then assigns priority to the channels for message scheduling. Here, a binary tree replication algorithm is proposed for message scheduling. To construct a forwarding set, this research calculates the probability of channel availability using the proposed routing algorithm. The simulation result shows that the proposed work achieves better performance in terms of the delivery ratio and overhead ratio compared to existing works. Here, routing is performed by a binary tree replication algorithm in which the routing process does not consider the trust value of the next hop, which leads to poor security, increases the data threat, and reduces routing performance.

The authors proposed hybrid beamforming for heterogeneous networks with single macro-base stations (MBS) and multiple small-base stations (SBS) [37]. Here, analog beamforming weight values are obtained by the eigendecomposition method. Digital beamforming weight values are optimized to increase the SINR of the channel. SUs share a similar frequency bandwidth to the MBS. Then, the weight values of the hybrid beamforming are calculated based on the signal transmission. Once the backhaul link weight values of the hybrid beamforming are estimated, they will will be used to forward the signal of the SU to the MBS. The simulation result shows that the proposed work achieves better performance in terms of computational complexity. Hybrid beamforming is performed to solve the computational complexity. However, this work does not consider beam characteristics (beam score and angle) and CSI for generating hybrid beams, which leads to inefficient beam generation that reduces the performance of hybrid beamforming. Hybrid beamforming was proposed with high precision for 5G mmWave communication in [38]. The proposed hybrid beamforming MIMO transceiver includes two main sections such as the mmWave active phased array and IF baseband subsystem. Every digitalized chain of the subsystems is connected with one phase sub-array with eight antennas. The proposed eight-bit phase shifter includes a digital step attenuator, 1-bit shifter, quadrature coupler, and power shifter, which are used for achieving a better linearity of a signal. This research analyzes the effect of RF chain errors on the final beamforming patterns, which are used for achieving high precision. The simulation result shows that the proposed work achieves better performance in terms of cost and data rate compared to the existing work. Here, eight-phase shifters are used for generating hybrid beamforming; however, they need to be selected optimally; otherwise, they lead to high power consumption, which will increase hardware complexity. Table 2 describes the summary of the existing methods.

## 3. Problem Statement

Autonomous driving faces many challenges such as spectrum sensing, security, and more energy consumption in edge-enabled 6G mmWave massive MIMO CRN-based IoCV environments. Existing studies address many issues such as scalability, spectrum scarcity, and mobility, which are addressed in research works discussed in this section. The major objective of this research is to maximize packet delivery ratio $\left(P_{d}\right)$, throughput $\left(T_{h}\right)$, and spectrum efficiency $\left(S_{e}\right)$ and minimize sensing delay, which is formulated as follows:

(1) $$\begin{array}{c} \begin{array}{c} Obj\leftarrow Max\sum_{i-1}^{n}P_{d},T_{h},S_{e} \end{array} \end{array}$$

(2) $$\begin{array}{c} \begin{array}{c} Max\;P_{d}=Arg\;O_{r}=Arg\left\{\sum_{i=1}^{n}\left(\tilde{\omega }\times SU_{i}\right)\right\} \end{array} \end{array}$$

where $O_{r}$ represents the optimized route and $\tilde{\omega }$ represents the binary value (0, 1), regardless of whether *SU* is in the optimal route or not. If $SU_{i}$ is selected as the next hop, then the value of $\tilde{\omega }$ is one; otherwise, it is zero:

(3) $$\begin{array}{c} \begin{array}{c} Max\;S_{e}=\;Max\sum_{i\in R}U_{i,e(i)} \end{array} \end{array}$$

where *H* represents the spectrum efficiency, U represents the spectrum utilization, and e(i) represents the spectrum utilization of SUs in the available spectrum in the channel. The other major problems of the existing approaches are explained below. The authors of [39] proposed a novel handoff method by selecting an optimal channel in the cognitive radio network (CRN). Carrier sense multiple access/collision avoidance (CSMA/CA) was used for spectrum sensing. The major problems of this research are listed as follows:

- Here, CSMA/CA is used for spectrum sensing, which uses a four-way handshaking process (RTS/CTS/DATA/ACK) for efficient sensing; however, it may sense efficiently, leading to additional traffic during the CTS handshaking process.
- The secondary users are coordinated with each other in CCC where they are provided with PCLs for every individual secondary user. They are inter-connected with the hop network, which is less secure and easy to attack.
- At the decision-making stage, secondary users have to broadcast their PCL list obtained from the DCF mode and then spectrum sensing using CSMA/CA is performed; however, this proposed work does not consider the trust factor that leads to less security. Hence, the attackers easily compromise the spectrum, leading to inefficient spectrum sensing.

The authors of [40] perform hybrid beamforming for the mmWave MIMO system, and channel decomposition is constrained. This work uses two-stage approaches for mitigating channel decomposition problems, which leads to multi-user interference (MUI) and intra-user interference. The authors of [41] propose a novel hybrid beamforming model to provide low complexity beams in frequency-selective channels. This work exploits both time and frequency domain characteristics for efficient beamforming. The major problems of this research are listed as follows:

- Here, hybrid beamforming is used to mitigate the channel decomposition, but channel state information (CSI) is not considered which leads to less efficient beams.
- Here, phase shifters used in the analog precoder are used to maintain the frequency range and allow the signal to be passed in the baseband, but the arrangement of phase shifters in the analog precoder is too hard to analyze. However, in the transmitter side, it causes more delay, which leads to inappropriate signal transmission.
- Here, more L taps are used to maintain signal power and reduce hardware complexity, and they are not easy to analyze. However, using more phase shifters leads to the time delay in the RF beamer side.
- Here, more RFs are used on the combiner side for reducing MUI and obtaining high SNR, which improves the beamforming performance overall. However, due to the increased usage of RFs, energy consumption is high, which leads to higher costs.

In paper [42], imperfect spectrum sensing is mitigated by the two-loop procedure (TLP) in wireless-powered cognitive-based mobile edge computing. To resolve the issue of imperfect spectrum sensing, the proposed work performed three stages including energy harvesting (EH), wireless power transfer (WPT) offloading, and spectrum sensing. The major problems of this research are listed as follows:

- Here, for spectrum sensing, secondary users have to wait until the primary transmitter responds with idle spectrum holes for sensing, which may lead to EH for secondary users but increased time consumption.
- Here, the mobility of the spectrum is not considered, which leads to spectrum scarcity during spectrum sensing; hence, it increases transmission delay.
- Here, partial offloading is used for wireless power transfer to the edge server. Initially, secondary users are allowed for local computing, which may lead to wrong spectrum sensing due to the dynamic environment.
- Here, single antenna assumptions are made for wireless power transfer and partial offloading, which may be easy to understand but in real-time scenarios; these assumptions may not be used, which leads to low antenna gain and less robustness.

The authors of [43] proposed a method of a two-hop routing algorithm for CR-VANET based on a multi-objective Harris Hawks optimization algorithm. The main problems of this research are defined as follows:

- Here, spectrum sensing and segment management are performed at the same time in SUs, and channel allocation is carried out by the RSU in parallel. It is effective, but it may lead to an unnecessary collision between SUs in a CR-VANET environment.
- Here, the optimal routing mobility of the vehicle is taken as a parameter by using two hop neighbors. It may be effective, but due to the dynamic mobility of vehicles in the CR-VANET environment, it leads to high routing overhead.
- Here, for the optimal routing direction, vehicle state, mobility, and channel availability are taken as parameters that give the optimal way to the destination by using a two-hop network. However, past information about the neighbors is not considered, which leads to inefficient path selections.

## 4. 6GCRN-IoCV System Model

**Figure 2:**
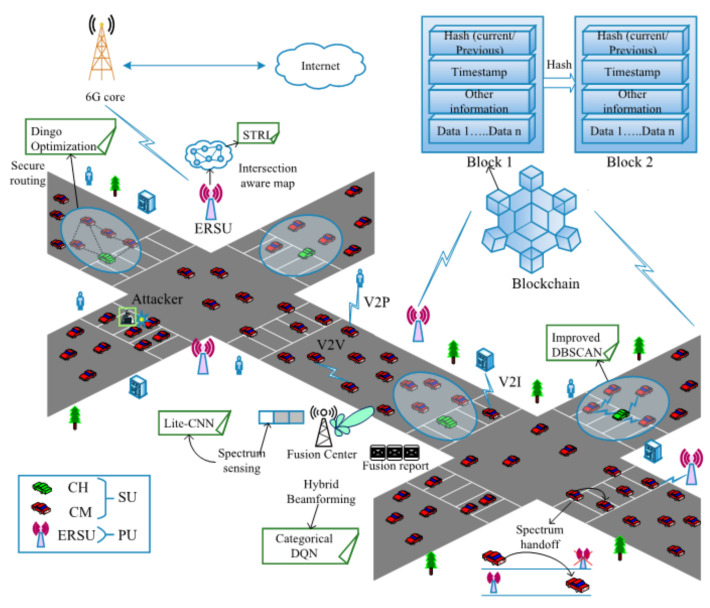
6GCRN-IoCV system model.

The proposed 6GCRN-IoCV system model includes n number of secondary users (SUs), which are known as vehicles, and n number of primary users (PUs), which are known as edge-assisted RSUs, in which edge computing is used to provide additional resources to the RSU for performing further processes such as routing and the collection of intersection information. All SUs can access the licensed spectrum and they send the sensing report to the fusion center (FC) in an encrypted manner, which enhances the security of the environment. FC is employed with an mmWave massive MIMO antenna. The network also consists of a malicious SUs, for which their job is to exploit the entire network by its malicious behavior, which results in less spectral efficiency and security threats. The malicious behavior can also affect the important network parameters such as the throughput, delay, and packet delivery ratio. Here, 6G communication is used for increasing the transmission speed, which increases the throughput during data transmission between IoCV. Spectral efficiency is achieved by proposing hybrid beamforming using massive MIMO in the CRN. Here, blockchain is used to store all transactions in the network. This research mainly focuses on spectrum efficiency and security during data transmission using spectrum sensing and secure routing in edge-enabled 6G mmWave massive MIMO CRN-based IoCV environments. Figure 2 illustrates the system model of the 6GCRN-IoCV method.

### 4.1. Network Management

Initially, all PUs, SUs, and pedestrians are authenticated by considering their PUF, ID, and location using blockchain to increase the security of the V2X environment. Here, ERSUs (edge-assisted RSUs) are considered to be PUs, and vehicles are considered to be SUs. To reduce energy consumption and latency, we propose ERSU. For managing the network, we perform clustering. Only authenticated users can be involved in the clustering process by considering direction, distance local density, and location by using the improved DBSCAN algorithm. In general, the DBSCAN algorithm discovers the clusters based on the density approach by a core point with minPts, epsilon (eps), and the classification of noise points. To improve the processing speed, the improved DBSCAN algorithm is proposed for performing clustering based on the similarity of the neighbor, i.e., the Euclidean distance is implemented for measuring the distance between the SUs and triangular inequality is considered to reduce the unwanted distance computations.

Initially, the eps parameter is derived by the Euclidean distance between the two SUs as R and T, and it is expressed as follows:

(4) $$\begin{array}{c} \begin{array}{c} d\left(R,T\right)=\sqrt{\sum_{k=1}^{j}{\left(r_{k}-t_{k}\right)}^{2}} \end{array} \end{array}$$

A minimum number of points is also known as minPts. The center of the neighbor SU (N) is defined by a set of points (P) that is nearest to N. The local density of the specific SU is derived by using the minPts, and it is expressed as follows:

(5) $$\begin{array}{c} n=\frac{minPts}{\max_{r\in P}d\left(n,r\right)} \end{array}$$

where *P* is N’s neighbor which consists of minPts data points that are closer to N, and *d(n,r)* represents the distance function. A greater local density of N determines a small local radius. To calculate the difference between the core point and density point, a different metric is defined, which is represented as follows:

(6) $$\begin{array}{c} {℧}_{N}=\min_{n_{r}>n}d\left(N,r\right) \end{array}$$

where ${℧}_{N}$ denotes the minimum distance of all SUs with high local density. The maximum distance is derived as follows:

(7) $$\begin{array}{c} {℧}_{N}=\max_{r\in d}d\left(N,r\right) \end{array}$$

To reduce redundant distance computations, triangular inequality is used between the SUs *R* and *T*, which is expressed as follows:

(8) $$\begin{array}{c} \left|R+T\right|\leq \left|R\right|+\left|T\right| \end{array}$$

After constructed clustering, the cluster head (CH) is elected based on maximum RSSI (v) and trust ($\tilde{o}$) for transmitting the data between the cluster members (CM). Every CM in the cluster transmits data to CH for further processing.

**Figure 3:**
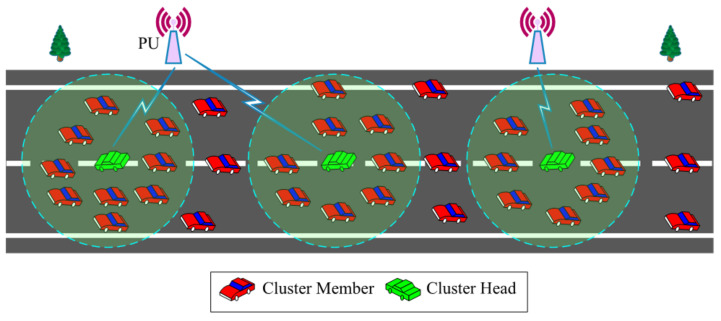
DBSCAN clustering of SUs.

The proposed network provides the intersection’s safety information to reduce the collision in the intersection. Intersectional information is collected by ERSU and stored in terms of past historical data. ERSU collects the following information from the vehicle: vehicle speed, destination, trajectory, RSSI, and location. Based on this information, ERSU generates an intersection-aware map. The intersection information is predicted by considering past historical data using the spatial and temporal-based logistic regression algorithm (STLR) based on the calculation of weights to reduce collisions in the network and tp efficiently manage the network, which is expressed as follows:

(9) $$\begin{array}{c} I_{m}=\log\frac{c}{1-c} \end{array}$$

where $I_{m}$ denotes the intersection-aware map, and c represents the intersection prediction probability. Figure 3 illustrates the density-based clustering of SUs. The improved DBSCAN-based clustering is explained in Algorithm 1.
**Algorithm 1:** Density-Based Clustering1:**Begin**2:**Input:**(minPts,eps,P)3:**Output:**(cluster formation)4:**for** each point p ∈ P **do**5: Mark p as visited6: *N*← Obtain Neighbors (p,eps)7: **If** $\left|N\right|$< minPts **then**8: Mark p as noise9: **Else**10: p → Cluster11: **for** each point p’ ϵ N **do**12:  N ← N/p’13:  **If** p’ not visited **then**14:  Mark p’ as not visited15:  p’ ← Obtain Neighbors (p’, eps)16:  **If**$\left|N^{\prime }\right|\geq minpts$ **then**17:  N ← N ⋃$N^{\prime }$18:  **If** p’ is not still a member of any cluster **then**19:  Cluster ← Cluster ⋃$\left\{p^{\prime }\right\}$20: **end for**21:**end for**22://CH Selection23:Compute $H_{c}$ = $\left\{v,\tilde{o}\right\}$24:Elect CH based on $H_{c}$25:**If**$\left(u==v\;\&\&\;\tilde{o}\;\right)$**then**26:Select u as CH27:**End**

### 4.2. Secure Spectrum Sensing and Mobility Management

Spectrum sensing is performed to increase spectrum utilization in the network. Each SU performs spectrum sensing and transmits the sensing report to the fusion center (FC) to address the problem of spectrum scarcity. In our study, SU encrypts the sensing report and then transmits the FC to increase security, which works against eavesdropping attacks in the network. Spectrum sensing is performed using the lightweight convolution neural network (Lite-CNN) by considering SNR, trust factor for every time interval, and noise level. The spectrum sensing by the SU is formulated as follows:

(10) $$\begin{array}{c} H_{1}:x\left(n\right)=r\left(n\right)+v\left(n\right) \end{array}$$

(11) $$\begin{array}{c} H_{0}:x\left(n\right)=v\left(n\right) \end{array}$$

where r(n) represents the signal, which is suffering from channel fading and path loss, and v(n) represents the Gaussian noise with zero means. $H_{1}$ and $H_{0}$ represent the presence and absence of PU, respectively, which are sensed by Lite-CNN and include five layers such as a convolutional layer, max-pooling layer, lite module layer, fully connected layer, dropout layer, and softmax layer. The softmax layer is the classifier that includes two possible classes, such as the PU’s presence and absence, which is denoted by $C_{i}$ and is formulated as follows:

(12) $$\begin{array}{c} \sum_{i}^{2}C_{i}=1 \end{array}$$

The overall input of the softmax layer is defined as follows:

(13) $$\begin{array}{c} P_{i}=\sum_{j}x_{j}w_{ji} \end{array}$$

where *x* represents the upper layer output, and *w* represents the weight value of the softmax. The calculation of the final output is defined as follows:

(14) $$\begin{array}{c} C_{i}=\frac{Exp(P_{i})}{\sum_{i}^{2}Exp\left(P_{i}\right)} \end{array}$$

The output class $O_{i}$ is defined as follows:

(15) $$\begin{array}{c} O_{i}=ArgmaxC_{i} \end{array}$$

(16) $$\begin{array}{c} O_{i}=ArgmaxP_{i} \end{array}$$

Here, cross-entropy is used for classifying the original output from the expected one. The calculation of cross-entropy is defined as follows:

(17) $$\begin{array}{c} T\left(a,b\right)=-\sum_{x}a\left(x\right)\log b\left(x\right) \end{array}$$

where T(a,b) represents cross-entropy, *a* represents the expected output, and b represents the original output. a(x)and b(x) represent a probability distribution. The filter counts are changed adaptively based on the input size. Table 3 illustrates the layers and filters of Lite-CNN.

Spectrum mobility management is an important process due to the mobile nature of vehicles and increasing transmission delay. Hence, we perform spectrum handoff while PU is active or busy. For that, SU selects the optimal channel by considering noise, SNR, channel availability, service time, congestion, and trust level. If SU detects the optimal channel, then we perform secure handoff, which reduces transmission delay and collision between PU and SU. Optimal channel selection-based handoff leads to high throughput. The probability of arrivals is calculated as follows:

(18) $$\begin{array}{c} P_{i}\left(\sigma \right)=\frac{{\left(\delta \sigma \right)}^{i}}{i!}e^{-\delta \sigma } \end{array}$$

**Figure 4:**
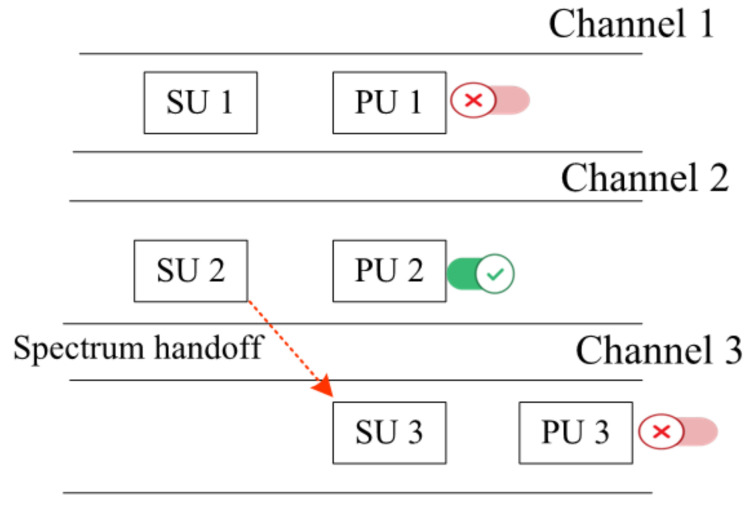
Spectrum handoff.

Based on the optimal channel selection, we calculate the possibility of spectrum handoff from one SU channel to another channel, which is defined as follows:

(19) $$\begin{array}{c} Pr\left(SC_{i}\left(\sigma \right)=0\right)<l_{p} \end{array}$$

where Pr represents the probability, $SC_{i}$ represents the state of the channel, and $l_{p}$ represents the probability limit value, which denotes that the corresponding channel is active, and SU should perform a handoff:

(20) $$\begin{array}{c} \left\{\begin{array}{c} Pr\left(Sc_{i}\left(\sigma \right)=0\right)\;\geq q_{t}\\ Pr(t_{i,off}>\varepsilon )\;\geq \tau \end{array}\right. \end{array}$$

where $q_{t}$ represents the limit of probability, and the current channel is considered inactive. ϵ=ρ+∝ is considered as the information period and time interval, and τ represents the limit of probability, and the channel is considered inactive. By performing secure spectrum sensing and handoff, we mitigate SSDF and PUF attacks in the environment. Figure 4 represents the proposed spectrum handoff techniques in which three channels are provided. In the first channel, PU 1 is inactive; thus, there is no need for spectrum handoff, while in the second channel, PU 2 is active; thus, SU 2 must select an optimal channel to perform spectrum handoff. Therefore, it performed handoffs to channel 3, where PU 3 is inactive.

### 4.3. Secure Routing

Optimal routing is an essential process in V2X to maintain communication between vehicles. Here, secure routing is performed by ERSU because it collects and stores the environment data. Initially, we performed the next forwarder selection by considering link stability, energy, trust, and relative velocity, which is carried out by SUs. By selecting the next forwarder, ERSU discovers the secure route by considering vehicle trajectory, the number of hops, vehicle speed, and link stability using the Dingo Optimization Algorithm (DOA), which selects secure routing for data transmission. The proposed DOA algorithm includes three processes: searching, encircling, and attacking. Initially, the dingoes detect the prey’s location and then trace the location. Here, ERSU is considered a dingo, and SUs are considered prey. The dingoes discover the prey for routing. The dingoes’ behavior is defined as follows:

(21) $$\begin{array}{c} d=\left|A.p\left(y\right)-\overset{´}{p}\left(i\right)\right| \end{array}$$

(22) $$\begin{array}{c} \begin{array}{c} \overset{´}{p}\left(i+1\right)=\left|p(i)-B.d\right| \end{array} \end{array}$$

(23) $$\begin{array}{c} A=2.\hat{a}1 \end{array}$$

(24) $$\begin{array}{c} B=2\dot{b}.\hat{a}2-\dot{b} \end{array}$$

(25) $$\begin{array}{c} \dot{b}=3-\left(\overset{´}{I}\times \left(\frac{3}{{\overset{´}{I}}_{max}}\right)\right) \end{array}$$

where d represents the distance between the prey and dingo; A and B represent the coefficients; $\overset{´}{p}$ and p represent the position of the dingo and prey, respectively. Based on the current prey position $\left({\overset{´}{p}}^{*},q^{*}\right)$, the dingoes also update their current position of $\left(\overset{´}{p},q\right)$. $\hat{a}1$ and $\hat{a}2$ represent the random vectors in [0, 1]. The agent does not discover the optimal position of the prey. The hunting plan of the dingoes includes beta, gamma, and other known prey locations. The beta dingo always performs hunting and another dingo also performs hunting. In this manner, hunting is performed to search for the best prey; thus, we consider the best values. According to the best search agent location, the remaining dingoes update their position. The mathematical representation for hunting is defined as follows:

(26) $$\begin{array}{c} d_{\beta }=\left|A_{1}.{\overset{´}{p}}_{\beta }-\overset{´}{p}\right| \end{array}$$

(27) $$\begin{array}{c} d_{\gamma }=\left|A_{2}.{\overset{´}{p}}_{\gamma }-\overset{´}{p}\right| \end{array}$$

(28) $$\begin{array}{c} d_{0}=\left|A_{3}.{\overset{´}{p}}_{0}-\overset{´}{p}\right| \end{array}$$

(29) $$\begin{array}{c} {\overset{´}{p}}_{1}=\left|{\overset{´}{p}}_{\beta }-B.d_{\beta }\right| \end{array}$$

(30) $$\begin{array}{c} {\overset{´}{p}}_{2}=\left|{\overset{´}{p}}_{\gamma }-B.d_{\gamma }\right| \end{array}$$

(31) $$\begin{array}{c} {\overset{´}{p}}_{3}=\left|{\overset{´}{p}}_{0}-B.d_{0}\right| \end{array}$$

The calculation of dingo intensity is defined as follows:

(32) $$\begin{array}{c} I_{\beta }=\log\left(\frac{1}{f_{\beta }^{-(1e-100)}}+1\right) \end{array}$$

(33) $$\begin{array}{c} I_{\gamma }=\log\left(\frac{1}{f_{\gamma }^{-(1e-100)}}+1\right) \end{array}$$

(34) $$\begin{array}{c} I_{0}=\log\left(\frac{1}{f_{0}^{-(1e-100)}}+1\right) \end{array}$$

If a position update is not performed, this will denote that the dingo completed hunting. If the B value is less than −1, then the prey moves from the current search agent. In this manner, ERSU selects an optimal and secure route for data transmission. By performing secure routing, we mitigate Sybil and black-hole attacks in the environment. The secure routing is explained in Algorithm 2.
**Algorithm 2:** Secure Routing1:Initialize $SU_{i}=SU_{1},SU_{2},\cdots ,SU_{n}$2:Initialize searching agent d3:Initialize $\dot{b},A,B$4:**While** not reaching termination conditions **do**5:Compute the fitness value for each dingo6:$d_{\beta }$ = First best search7:$d_{\gamma }$ = Second best search8:$d_{0}$ = Other search results9:I = 110:Repeat11:**for** i = 1:d **do**12: Update the status of the current search agent13:**end for**14:Compute the fitness value for each dingo15:Calculate the value of $\dot{b},A,B$16:I = I + 117:**if** (I > Stopping condition) **then**18:Optimal route selection is performed19:**End if**20:**End While**

### 4.4. Secure Hybrid Beamforming

Beamforming is performed to enhance the signal’s quality, which improves transmission speeds. However, only analog and digital beamforming leads to high hardware complexity and spectrum inefficiency due to the high amount of RF chains. To overcome these issues, we perform hybrid beamforming, which provides computational efficiency, hardware efficiency, and spectral efficiency. For hybrid beamforming, we propose a categorical DQN in which DQN has multiple agents for enhancing performance. The multi-agent categorical DQN exploits the Markov decision process, and three agents are involved in taking actions $\left(m_{t}\right)$ at time t based on the environment, which can be represented as $m_{t1},m_{t2},m_{t3}\in M$. The actions are taken at the state $\left(sT_{t}\right)$, and for every successful interaction, each agent receives rewards $\left(re_{t}\right)$. The multi-agent categorical DQN aims to discover the policy $\left(P^{⌀}\right)$ in which all agents can be focused to complete the task. $P^{⌀}$ should be maximized to obtain the expected yields, which can be formulated as follows:

(35) $$\begin{array}{c} P=arg\max_{P^{⌀}}E^{p}P^{{⌀}^{\left(H\right)}}\left[R(H)\right] \end{array}$$

where $E^{p}P^{{⌀}^{\left(H\right)}}$ is the expected yield, H represents the history of the time, and R(H) denotes the sum of the histories at a time for providing instantaneous rewards.

The first agents with the action m $m_{t1}$ designate hybrid beamforming design, i.e., analog and digital precoders, and analog and digital combiners. Analog precoding and digital precoding are performed in ERSU (PU) in which analog precoder $\left(F^{RF}\right)$ is carried out in the time domain and digital precoder $\left(F^{BB}\right)$ is carried out in the frequency domain. The normalization of the analog precoder is carried out because it fulfills the constrictions of the constant modulus as it is realized by the phase shifters in the network. The normalized analog precoder is denoted as follows:

(36) $$\begin{array}{c} {\left|{\left[F^{RF}\right]}_{i,j}\right|}^{2}=1 \end{array}$$

The transmitted total power $\left(Tr_{totl}\right)$ is acquired by the coupling of $F^{RF}$ and $F^{BB}$, which can be formulated as follows:

(37) $$\begin{array}{c} \sum_{k=1}^{k}{∥F^{RF}F^{BB}\left[k\right]∥}^{2}\leq Tr_{totl} \end{array}$$

where *k* denotes the subcarriers. The combing of analog precoding and digital precoding results is performed at the fusion center by the analog and digital combiner. Initially, analog combiner $\left(W^{RF}\right)$ combines the received signal in the analog domain, and the cyclic prefix by the hybrid precoder is removed and provided to the digital combiner $\left(W^{BB}\right)$ in the frequency domain. The overall analog–digital precoding and analog–digital combining of the received signal matrix can be represented as follows:

(38) $$\begin{array}{c} y\left(k\right)=W_{G}^{R}\left[k\right]G\left[k\right]F^{E}\left[k\right]s\left[k\right]+W_{G}^{R}\left[k\right]n\left[k\right] \end{array}$$

where the following is the case

(39) $$\begin{array}{c} F^{RF}F^{BB}\left[k\right]=F_{E}\left[k\right] \end{array}$$

(40) $$\begin{array}{c} W^{RF}W^{BB}\left[k\right]=W_{G}\left[k\right] \end{array}$$

where G[k]denotes the matrix of the channel among the fusion center and PUs; n[k] is the noise, i.e., additive white Gaussian noise; and s[k] represents the data symbols at each subcarrier.

The second agent with action $m_{t2}$ predicts optimal N-taps to increase performance in terms of signal power and time. The N-taps are designed for $W^{RF}$ (RF beamformer). Usually, N-taps represent the phase shifters (matched filters) and delay lines *lin, lin*∈$\left\{1,\cdots ,N-1\right\}$. The outputs of the N-taps are connected to the adder circuit. The RF beamformer can be represented as follows:

(41) $$\begin{array}{c} \begin{array}{c} W^{RF},lin\;A\;PUs=\frac{1}{\sqrt{PUse^{j\theta linAPus}}} \end{array} \end{array}$$

WRF, lin A PUs = 1PUsejlin A Pus

From the above equation, the several antennas of $A\in \left\{1,\cdots ,A\right\}$ PUs refer to ERSUs. The combiner side impulse response of the N-tap RF beamformer can be formulated as follows:

(42) $$\begin{array}{c} W^{RF}\left(n\right)=\sum_{lin=-N+1}^{0}W^{RF},lin\;\delta \left(n-lin\right) \end{array}$$

The N-tap beamformer noise n[k] and channel matrix G[k] can be formulated as follows:

(43) $$\begin{array}{c} \left\{\begin{array}{c} G\left[k\right],\;N-tap\left(n\right)\;=\sum_{lin=-N+1}^{lin=N-1}\;W_{n-lin}G\left(lin\right)\\ n\left[k\right],\;N-tap\left(n\right)\;=\sum_{lin=-N+1}^{0}\;W_{n-lin}n\left(lin\right) \end{array}\right. \end{array}$$

Here, hybrid beams are generated based on the CSI, beam score, the direction of the angle, array factor, elevation angle, and azimuth angle. CSI is predicted based on RSSI, SINR, spectral efficiency, and environmental factors (weather, temperature, and humidity).

A third agent with action $m_{t3}$ is used for collecting and updating the feedback from the users. Hence, the elevation angle and azimuth angle are predicted by obtaining feedback from the users. The azimuth angle and elevation angle are calculated during multipath propagation, which can be formulated as follows:

(44) $$\begin{array}{c} azm_{An}\left(\theta ,⌀\right)=\left[1,e^{j2\pi d/\sigma \;cos\left(\theta \right)sin\left(⌀\right)\cdots }e^{j2\pi \left(A-1\right)dis/\sigma \;cos\left(\theta \right)sin\left(⌀\right)}\right] \end{array}$$

(45) $$\begin{array}{c} ele_{An}\left(⌀\right)=\left[1,e^{j2\pi d/\sigma \;cos\left(\theta \right)sin\left(⌀\right)\cdots }e^{j2\pi \left(A-1\right)dis/\sigma \;cos\left(\theta \right)sin\left(⌀\right)}\right] \end{array}$$

From the above equations, σ represents the wavelength, and *dis* denotes the distance between elements.

The three agents $m_{t1},m_{t2}$, and $m_{t3}$ perform actions based on the states of combining analog and digital beamforming, N-tap selection, and user feedback, respectively. The value function of the action can be represented as follows:

(46) $$\begin{array}{c} Q^{⌀}\left(sT_{t},m_{t}\right)=E^{p_{P^{⌀}}\left(H\right)}\left[R\left(H\right)\right]sT_{1}=sT,m_{1}=m \end{array}$$

From the above equation, sT and *m* are the opening values of the state and action. The categorical DQN exploits the CNN to estimate the value function. The actions $m_{t1},m_{t2}$, and $m_{t3}$ are selected based on time t, and $Q^{⌀}$ ranks the actions. Finally, γ (the policy of greed) is engendered, which is represented as follows:

(47) $$\begin{array}{c} ⌀\leftarrow \left\{\begin{array}{c} 1-\gamma +\gamma /|Sc|,\;if\;m\;=\;arg\;\max_{m^{\prime }\in M}Q^{⌀}\left(sT,m^{\prime }\right)\\ \gamma /|Sc\;,\;otherwise \end{array}\right. \end{array}$$

where |Sc| is the action space scale of *M*. Reward $re_{t}$ is provided for successful hybrid beamforming. All information is retried by blockchain to produce secure hybrid beamforming. The beamforming factors are listed as follows:

***(i) Beam score (Bes)***—The generation of beams based on the scores, which can be formulated as follows:

(48) $$\begin{array}{c} Bes=\sum_{i=0}^{X}ch_{base}\times Ant_{Ve} \end{array}$$

where $Ant_{Ve}$ denotes the vector of an antenna with CSI information and past information, and $ch_{base}$ represents the baseband channel.

***(ii) Array Factor (*****${\mathrm{Ar}}_{\mathrm{fac}}$*)***—The position of the antenna in an array manner for determining its function is known as $Ar_{fac}$, which can be formulated as follows:

(49) $$\begin{array}{c} Ar_{fac}=\sum_{z=0}^{z-1}\sum_{v=0}^{v-1}\nabla_{zv}e^{j[z\left(DOA_{1}+\phi_{1}\right)+v\left(DOA_{2}+\phi_{2}\right)]} \end{array}$$

where $\phi_{1}$ and $\phi_{2}$ represent the radiation factor, $DOA_{1}$ and $DOA_{2}$ represent the direction of the angle location of the SUs, and $\nabla_{zv}$ represents the value of the antenna array’s weight.

***(iii) RSSI***—The ratio of the transmitted power to the received power for determining the total received signal power, which can be formulated as follows,

(50) $$\begin{array}{c} RSSI=\frac{Po^{t}}{Po^{r}} \end{array}$$

***(iv) SINR***—This is defined as the summation of interference and noise power from the power of the signal, which can be formulated as follows:

(51) $$\begin{array}{c} SINR=\frac{Po^{s}}{Po^{noi}+\sum_{i=0}^{X}Po^{inter}} \end{array}$$

From the above equation, $Po^{s}$ denotes the signal power, $Po^{inter}$ denotes the interference power, and $Po^{noi}$ denotes noise power.

***(v) Spectral efficiency (*$S_{e}$*)***—$S_{e}$ is defined as the ratio of the information rate to the channel bandwidth for determining the amount of transmitting information in the channel, which can be expressed as follows:

(52) $$\begin{array}{c} S_{e}=\frac{Info_{R}}{Band_{ch}} \end{array}$$

where $Band_{ch}$ denotes the channel’s bandwidth, and $Info_{R}$ is the rate of information.

**Figure 5:**
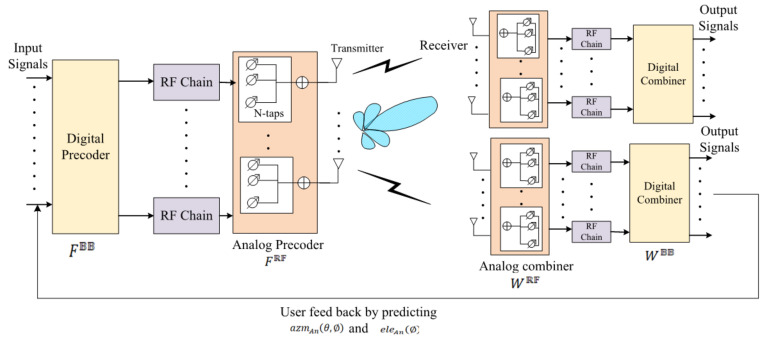
Multi-agent-based hybrid beamforming.

***(vi) Humidity (*Z*)***—The fading and scattering effects caused by the environment, which affect the transmission of a signal. Z can be formulated as follows:

(53) $$\begin{array}{c} Z=Z_{0}+Z_{wat} \end{array}$$

where $Z_{wat}$ denotes the humidity in water, which is represented in the unit of g· kg ${}^{-1}$, and $Z_{0}$ denotes the dry air attenuation which is represented in the unit of dB/m. Figure 5 denotes the multi-agent-based secure hybrid beamforming.

## 5. Experimental Results

In this section, the experimentation of the proposed 6GCRN-IoCV method is examined for managing the spectrum mobility and traffic in the network. This section is further divided into sub-sections including the simulation setup, application scenario, comparative analysis, and research summary, which are described below.

### 5.1. Simulation Setup

The proposed 6GCRN-IoCV method exploits edge technology and blockchain technology for secure spectrum access, routing, and hybrid beamforming in mmWave massive MIMO. This study is simulated by using simulation tools such as the simulation of urban mobility (SUMO) and an objective modular network that is tested in C++ (OMNeT++). The software and hardware configurations used for simulating the proposed work are the processor of Intel(R) Core(TM) i5-4590S CPU @ 3.00GHz 3.00 GHz, the operating system used is Windows 10 Pro, and the network simulators used are SUMO and OMNeT++. The hardware configuration includes a random access memory capacity of 6 GB and a hard disk capacity of 300 GB. Table 4 presents the simulation configuration used for simulating the proposed work.

### 5.2. Application Scenario (IoCV)

**Figure 6:**
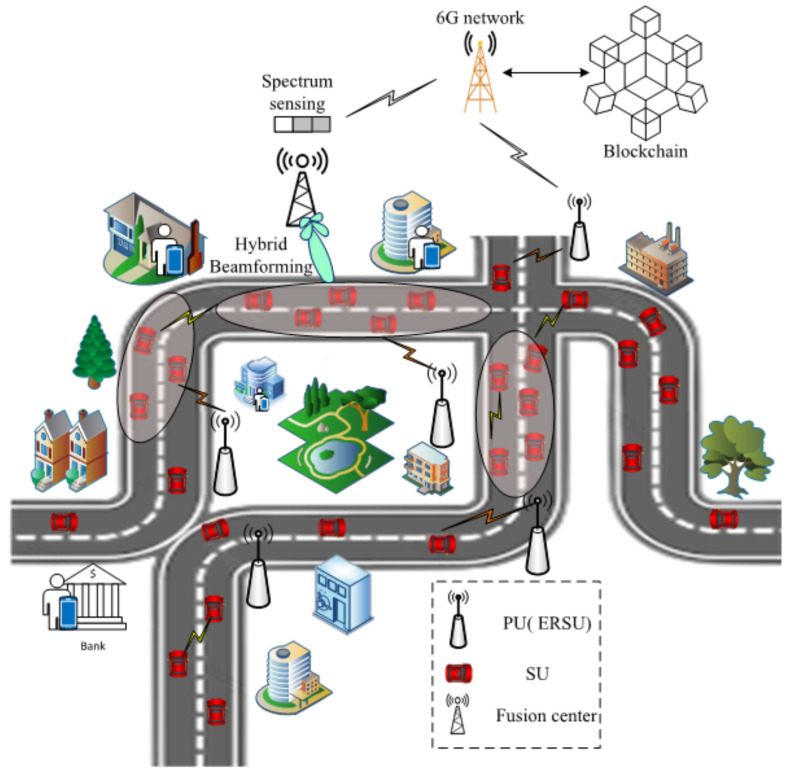
Use-case scenario.

The proposed 6GCRN-IoCV method is tested in an Internet of Connected Vehicles (IoCV) environment. IoCV is used for sharing emergency information, parking availability, intersection safety, traffic-related information, sharing audio files and video files, etc. As the number of IoCV increased over the years in the environment, the bandwidth requirements for the cognitive radio networks (CRN) grew in demand; CRNs were introduced to fulfill the needs of IoCV. In addition to that, security constraints also need to be resolved; thus, blockchain technology is also introduced in IoCV, and 6G communication is used to reduce communication overhead. Figure 6 represents the use case scenario of the proposed 6GCRN-IoCV in the IoCV environment. From the figure, it is shown that the vehicles in IoCV are assisted by PUs (ERSU). Each vehicle (SUs) contains onboard units (OBU) that are used for V2V and V2X 6G communications. These communications include emergency message sharing, file sharing, communications for entertainment purposes, etc. Once the spectrum is found to be available, the fusion center fuses the user request and performs hybrid beamforming, which enhances communication reliability. All vehicle information and environment information are stored in the blockchain.

### 5.3. Comparative Analysis

In this section, we carry out a comparison of the proposed 6GCRN-IoCV method with various existing methods, such as the NPH-CRN [39], HBF-MIMO [40], MEC-ISS [42], and CR-VANET [43] methods in order to evaluate the method’s performance. Numerous performance metrics are considered for analyzing performance such as the throughput, packet delivery ratio, delay, routing overhead, route acquisition delay, the probability of detection, spectral efficiency, total transmit power, and sensing delay, which are divided into three major categories: QoS analysis, spectrum efficiency analysis, and spectrum-sensing analysis. The definitions of these metrics are described below.

***(i) Throughput:*** It is defined as the amount of data delivered for each SU, which are located in the current network. It is measured by the ratio of the data size to the amount of time taken to transmit the data, which is expressed as follows:

(54) $$\begin{array}{c} T_{h}=\frac{Ʊ}{\overset{ˇ}{y}} \end{array}$$

where ℧ represents the data size, and $\overset{ˇ}{y}$ denotes the time needed for data transmission.

***(ii) Packet Delivery Ratio:*** This metric is calculated using the ratio of the number of packets delivered to the number of packets transmitted from the source user to the destination user, which is expressed as follows:

(55) $$\begin{array}{c} P_{d}=\frac{P_{r}}{P_{t}} \end{array}$$

where $P_{r}$ denotes the delivered packets, and $P_{t}$ represents the packets that are transmitted.

***(iii) Delay:*** This metric is measured by the amount of time taken between the source and destination to transmit the data. The delay is calculated by the sum of the queuing time (Q), propagation time (β), and transmission time (ψ). This is expressed as follows:

(56) $$\begin{array}{c} \underset{’}{z}=\left[Q+\beta +\psi \right] \end{array}$$

***(iv) Routing Overhead:*** This metric is used to calculate the routing overhead by the ratio of the total number of packets generated for route selection $\left(R_{se}\right)$ to the total number of transmitted packets $\left(\overset{ˇ}{t}\right)$, which is expressed as follows:

(57) $$\begin{array}{c} \tau =\frac{R_{se}}{\overset{ˇ}{t}} \end{array}$$

***(v) Route Acquisition Delay:*** It states the total time required between the source and destination nodes to transmit the route request message and receive the route replay message.

***(vi) Probability of Detection:*** This metric is used to measure the performance of FC by detecting and mitigating attacks efficiently.

***(vii) Total Transmit Power:*** This is defined as the amount of power required by the SUs to transmit data to the PU based on a number of hops.

***(viii) Sensing Delay:*** Each SU present in the network requires some time for spectrum sensing. This metric is used to calculate the total time taken by the SUs to sense the spectrum. It is measured by the ratio of the number of sensed channels to the number of packets transmitted by SUs, and it is expressed as follows:

(58) $$\begin{array}{c} \overset{ˇ}{D}=\frac{sc}{tp} \end{array}$$

where $\overset{ˇ}{D}$ denotes the sensing delay, *sc* represents the number of sensed channels, and *tp* shows the number of transmitted packets.

### 5.4. QoS Analysis

QoS is improved by performing data transmission with low packet loss and latency with a high packet delivery ratio and throughput. To evaluate the QoS of the proposed 6GCRN-IoCV method, several metrics are analyzed, which are listed below.

#### 5.4.1. Impact of the Throughput

**Figure 7:**
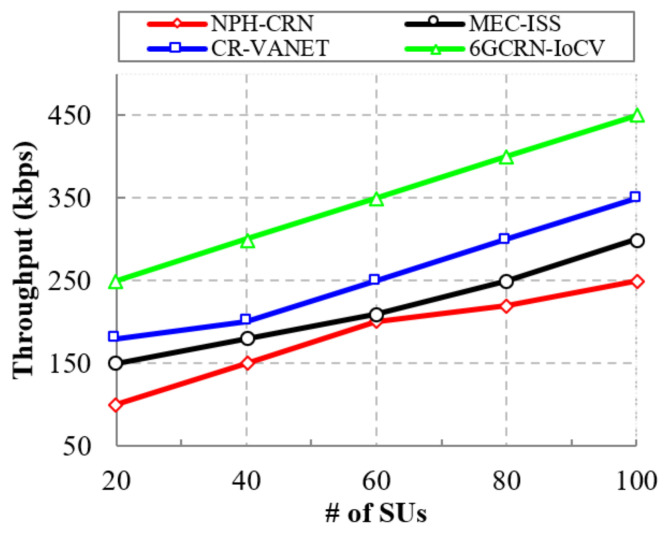
Throughput vs. the number of SUs.

A network with high throughput improves the QoS. Figure 7 represents the comparison of throughput to the number of SUs for the proposed 6GCRN-IoCV method and several existing methods. The throughput is increased by increasing the number of SUs. In the NPH-CRN method, communication is performed using CCC, which increases the security threats. In the MEC-ISS method, spectrum sensing is performed with a lack of spectrum mobility, which increases the latency and decreases the throughput. To overcome these issues, it is necessary to secure spectrum sensing and consider spectrum mobility during spectrum sensing in order to increase the throughput.

**Figure 8:**
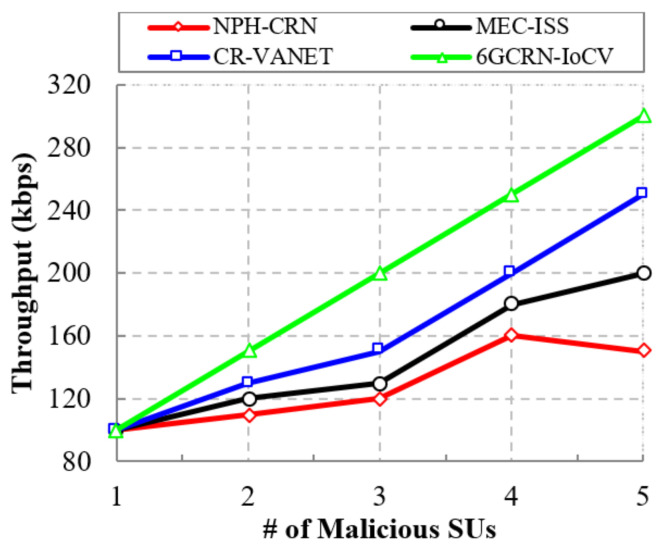
Throughput vs. the number of malicious SUs.

The result shows that the proposed 6GCRN-IoCV method achieves a high throughput of 450 kbps, which is 200 kbps greater than the NPH-CRN method, 150 kbps greater than the MEC-ISS method, and 100 kbps greater than the CR-VANET method. Similarly, Figure 8 illustrates the comparison of the throughput with respect to the number of malicious nodes. In the proposed 6GCRN-IoCV method, the clustering of authenticated SUs is performed to mitigate malicious nodes. The proposed work achieves 300 kbps of throughput, which is 150 kbps greater than the NPH-CRN method, 100 kbps greater than the MEC-ISS method, and 50 kbps greater than the CR-VANET method.

#### 5.4.2. Impact of the Packet Delivery Ratio

**Figure 9:**
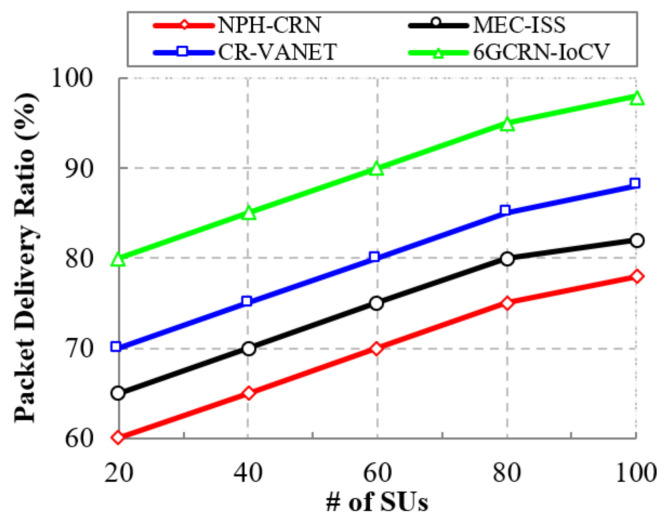
Packet delivery ratio vs. the number of SUs.

A decrease in packet loss increases the packet delivery ratio. Figure 9 illustrates the comparison of the packet delivery ratio to the number of SUs for the proposed 6GCRN-IoCV method and existing methods. Increasing the number of SUs increases the packet delivery ratio. In the CR-VANET method, routing is performed based on two-hop neighbors but the lack of consideration for the mobility environment increases routing overhead, which leads to high packet loss. In the proposed 6GCRN-IoCV method, various parameters are considered for secure routing using the DOA algorithm to reduce the packet loss, which increases the packet delivery ratio.

**Figure 10:**
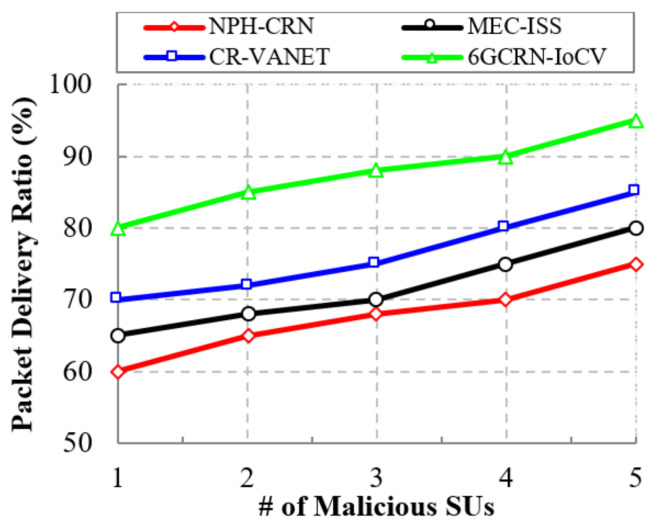
Packet Delivery Ratio vs. the number of malicious SUs.

The proposed 6GCRN-IoCV method achieves a high packet delivery ratio of 98% with a 20% difference with the NPH-CRN method, a 16% difference with the MEC-ISS method, and a 10% difference with the CR-VANET method. Similarly, Figure 10 shows the comparison of the packet delivery ratio to the number of malicious nodes. The proposed 6GCRN-IoCV method increases the security of the sensing report by performing encryption before transmitting the report to the FC to protect the report from malicious nodes. The result shows that the proposed 6GCRN-IoCV method has a maximum packet delivery ratio of 95%, whereas the maximum packet delivery ratio for NPH-CRN is 75%, and it is 80% for MEC-ISS and 85% for CR-VANET.

#### 5.4.3. Impact of the Delay

**Figure 11:**
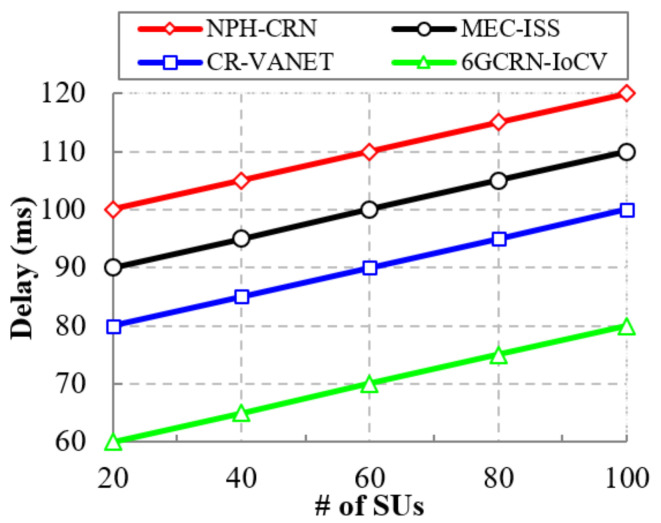
Delay vs. the number of SUs.

High spectral efficiency reduces the data transmission time, which decreases the delay. Figure 11 illustrates the comparison of the delay based on the number of SUs to the proposed 6GCRN-IoCV method and existing methods. Increasing the number of SUs increases the delay. In the existing methods, spectrum sensing was performed without considering the mobility environment and inaccurate spectrum sensing increases transmission delay. To reduce delay, spectrum sensing was performed using the Lite-CNN algorithm as well as the selection of the optimal channel to reduce spectrum scarcity, which increases transmission rate and, thereby, reduces delay.

**Figure 12:**
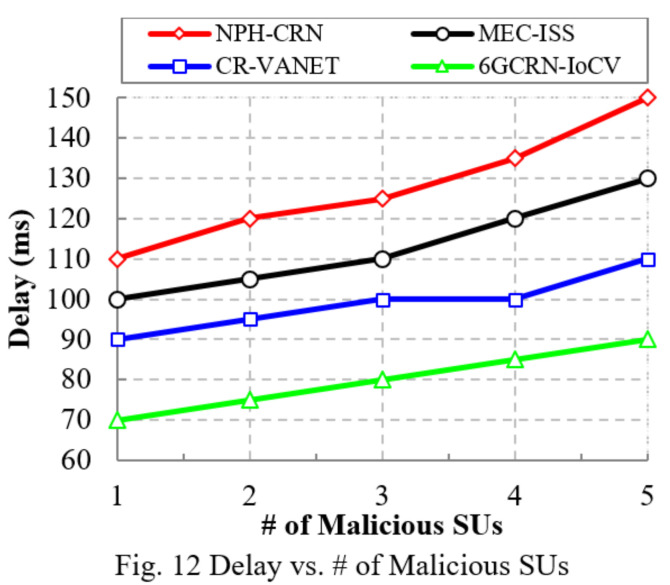
Delay vs. the number of malicious SUs.

The proposed 6GCRN-IoCV method attains low delay, which is 40 ms lower than the NPH-CRN method, 30 ms lower than the MEC-ISS method, and 20 ms lower than the CR-VANET method. The comparison of the delay with respect to the number of malicious SUs is the same as the number of SUs, which is illustrated in Figure 12. We can reduce the delay in the proposed 6GCRN-IoCV method by performing secure spectrum sensing using the Lite-CNN algorithm and considering the trust level of every time interval with a secure handoff to mitigate various attacks and reduce transmission delay efficiently. The proposed 6GCRN-IoCV model achieves a low delay of 90 ms with a 60 ms difference from the NPH-CRN method, 40 ms difference from the MEC-ISS method, and 20 ms difference from the CR-VANET method.

#### 5.4.4. Impact of the Routing Overhead

**Figure 13:**
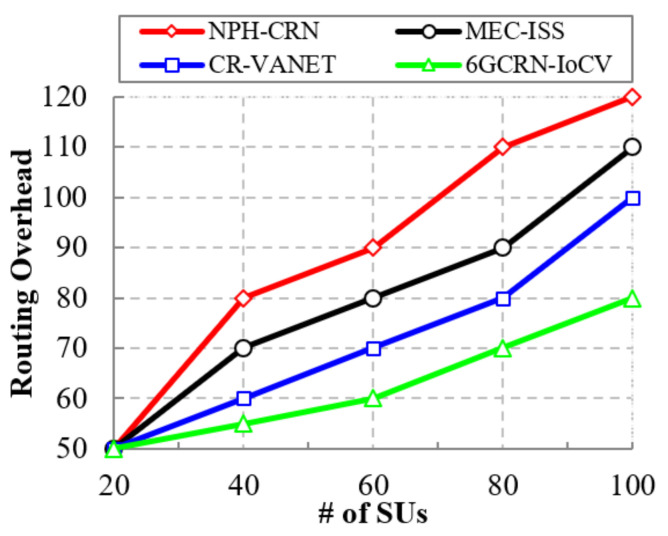
Routing overhead vs. the number of SUs.

Effective routing reduces the routing overhead, thereby minimizing the packet loss. Figure 13 shows the comparison of the routing overhead to the number of SUs between the proposed 6GCRN-IoCV method and several previous methods. The routing overhead is increased by increasing the number of SUs. In the NPH-CRN method, CSMA/CA was implemented to perform spectrum sensing with CTS handshaking, which leads to high traffic during the process of handshaking. In the CR-VANET method, insufficient hopping (i.e., two-hop) routing is performed in a dynamic environment that increases routing overhead.

In the proposed 6GCRN-IoCV method, secure spectrum sensing and routing are performed by considering several metrics to reduce routing overhead. This efficiently mitigates SSDF and PUF attacks. The difference in the routing overhead between the proposed 6GCRN-IoCV method and the NPH-CRN method is 40, and the difference in the routing overhead between the proposed method with the MEC-ISS and CR-VANET methods is 30 and 20, respectively.

#### 5.4.5. Impact of the Route Acquisition Delay

**Figure 14:**
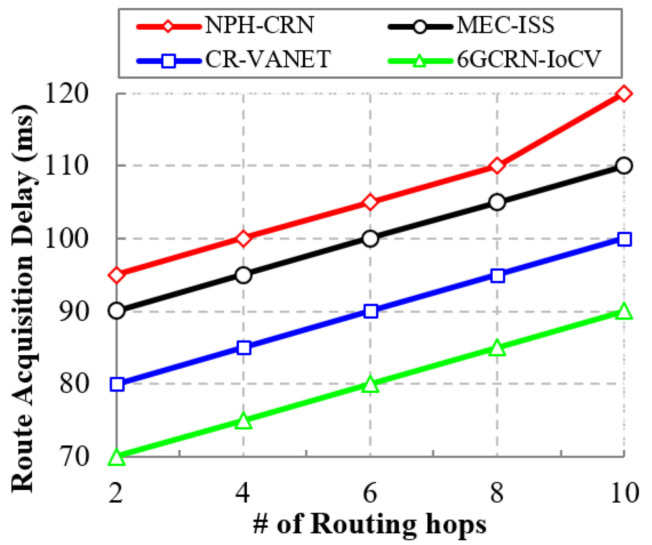
Routing acquisition delay vs. the number of routing hops.

An efficient routing path with enough spectrums reduces the route acquisition delay. Figure 14 illustrates the comparison of the route acquisition delay with the number of routing hops for the proposed 6GCRN-IoCV method and several previous studies. The route acquisition delay is increased by increasing the number of routing hops. In the NPH-CRN method, CCC is used for communication between SUs; however, it increases network traffic. Moreover, in the CR-VANET method, two-hop routing was performed with inefficient path selection, which increases route acquisition delay.

In the proposed 6GCRN-IoCV method, spectrum sensing and routing are performed by considering multiple parameters with high security to reduce the route acquisition delay when compared with the previous works. The proposed 6GCRN-IoCV method achieves a low route acquisition delay of 90 ms, which is 30 ms slower than the NPH-CRN method, 20 ms slower than the MEC-ISS method, and 10 ms slower than the CR-VANET method.

### 5.5. Spectrum Efficiency Analysis

Spectrum efficiency is achieved in the proposed 6GCRN-IoCV method by performing data transmission over a certain amount of channel bandwidth without spectrum wastage and spectrum scarcity. The evaluation of spectrum efficiency is performed by certain metrics, which are analyzed below.

#### 5.5.1. Impact of the Probability of Detection

**Figure 15:**
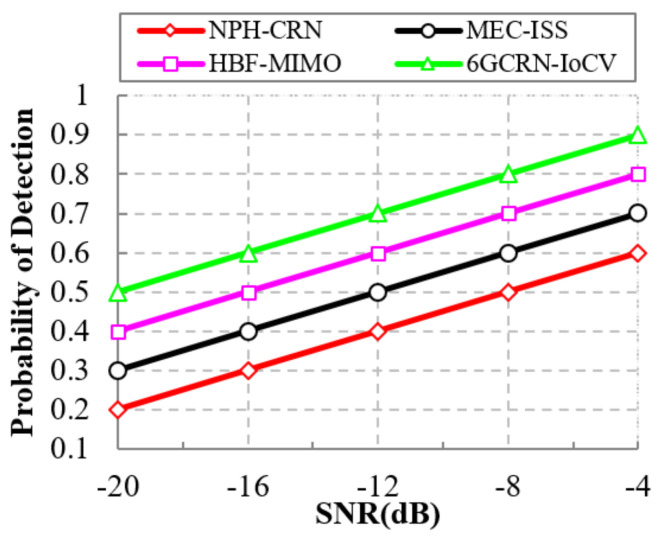
Probability of detection vs. SNR.

High detection probability leads to efficient data transmission with high spectrum security. Figure 15 illustrates the comparison of the probability of detection to the SNR value for the proposed 6GCRN-IoCV method and several existing methods. Increasing SNR increases detection probabilities. In the NPH-CRN method, the communication between the PU and SUs was performed using CCC with an inter-connected hop network that increases the security threats due to low detection probability. Various attacks are detected and mitigated in the proposed 6GCRN-IoCV method by performing secure spectrum sensing by encrypting the sensing report and secure routing based on the trust factor. This increases the detection probability of the proposed 6GCRN-IoCV method when compared with the previous works. The comparative result shows that the proposed 6GCRN-IoCV method achieves a detection probability of 0.9, which is 0.3 greater than the NPH-CRN method, 0.2 greater than the MEC-ISS method, and 0.1 greater than the HNF-MIMO method.

#### 5.5.2. Impact of the Spectral Efficiency

**Figure 16:**
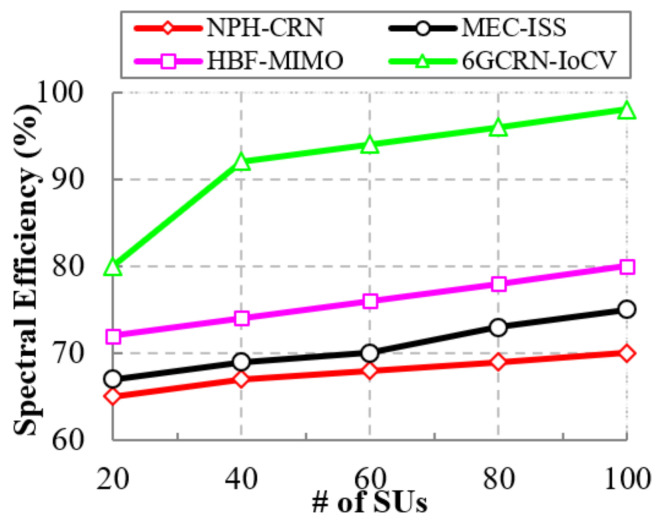
Spectral efficiency vs. the number of SUs.

The efficient utilization of the spectrum reduces the spectrum scarcity, which increases spectral efficiency. Figure 16 shows the comparison of spectral efficiency to the number of SUs between the proposed 6GCRN-IoCV method and several previous methods. Spectral efficiency increased with an increasing number of SUs. In the NPH-CRN method, CSMA/CA was used to perform spectrum sensing without knowing the trust factor that increases the network traffic when handshaking, which increases energy consumption. In the HBF-MIMO method, hybrid beamforming was used with more phase shifters to minimize the utilization of energy, which increases latency. These processes decrease the spectral efficiency. To overcome these problems, the sensing of spectrums is carried out using Lite-CNN and in consideration of a low number of phase shifters to reduce energy consumption, thereby increasing spectral efficiency. The proposed 6GCRN-IoCV method achieves a high spectral efficiency of about 98%, which is 28% higher than the NPH-CRN method, 23% higher than the MEC-ISS method, and 18% higher than the HBF-MIMO method.

### 5.6. Spectrum Sensing Analysis

Efficient and secure spectrum sensing decreases the delay and power utilization. The analysis of spectrum sensing is performed by several metrics, which are listed below.

#### 5.6.1. Impact of the Total Transmit Power

**Figure 17:**
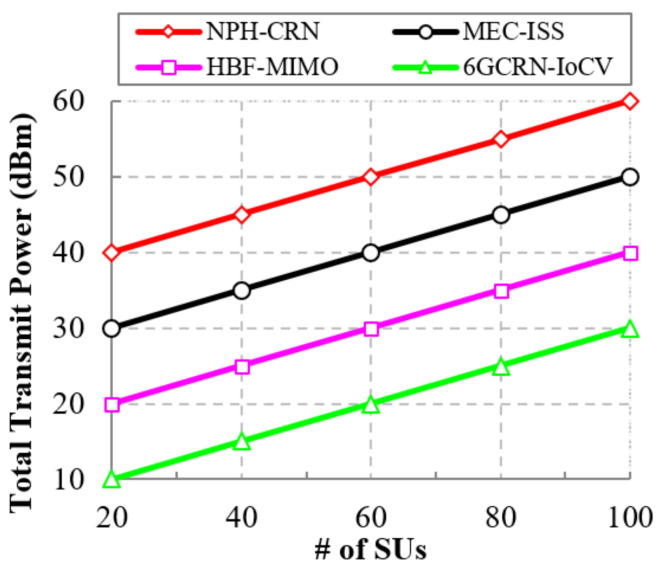
Total transmit power vs. the number of SUs.

Data transmission with low latency and high security reduced the total efficiently transmitted power. Figure 17 shows the comparison of the total transmit power to the number of SUs for the proposed and existing methods. The total transmit power increases with an increase in the number of SUs.

For data transmission, a common channel is used in the NPH-CRN method between the PU and SUs. However, it increases traffic, which increases the transmit power. In the HBF-MIMO method, to reduce the utilization of energy, phase shifters were implemented in an analog precoder. However, this analog precoder increases transmission delays, which increases transmission power. In the proposed 6GCRN-IoCV method, secure data transmission with a high transmission rate is securely performed by density-aware clustering and spectrum sensing in order to reduce transmission power. The difference in the total transmitted power between the proposed method and the NPH-CRN method is 30 dBm, and the difference between the proposed 6GCRN-IoCV method and the MEC-ISS and HBF-MIMO methods is 20 dBm and 10 dBm, respectively.

#### 5.6.2. Impact of the Sensing Delay

**Figure 18:**
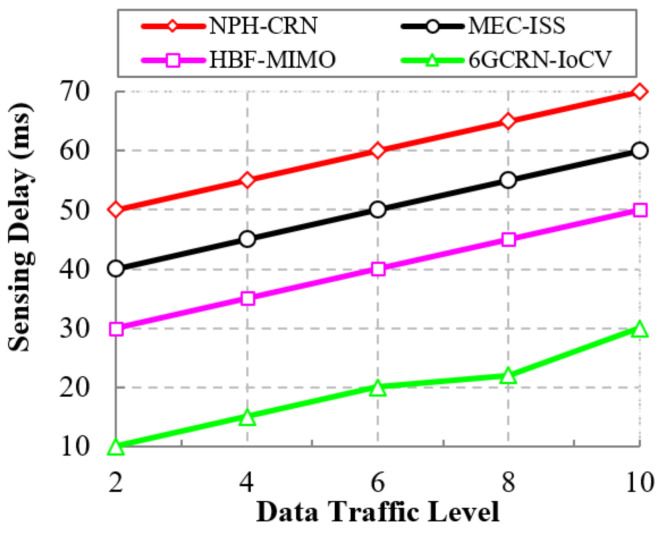
Data traffic level vs. sensing delay.

A low sensing delay provides efficient spectrum sensing to improve the routing performance. Figure 18 represents the comparison of the sensing delay to the data traffic level between the proposed 6GCRN-IoCV method and several existing methods. Sensing delay increases with an increase in data traffic levels. The NPH-CRN method performs communication with high data traffic using the common channel for the PU and SUs. In the MEC-ISS method, spectrum sensing is performed for PU and SUs sequentially, which increases the sensing delay. In the proposed 6GCRN-IoCV method, spectrum sensing is performed by considering various metrics such as SINR, trust factor, etc., using the Lite-CNN algorithm to reduce sensing delay. The analytical result illustrates that the proposed 6GCRN-IoCV method achieves a low sensing delay of a maximum of 30 ms, which is 40 ms slower than the NPH-CRN method, 30 ms slower than the MEC-ISS method, and 20 ms lower than the HBF-MIMO method.

### 5.7. Research Summary

This section explains the performance of the proposed 6GCRN-IoCV approach. Table 5 illustrates the numerical analysis of the existing and proposed approaches. Figure 7, Figure 8, Figure 9, Figure 10, Figure 11, Figure 12, Figure 13, Figure 14, Figure 15, Figure 16, Figure 17 and Figure 18 illustrate the comparison of performance metrics. The proposed work achieved a high throughput and packet delivery ratio due to effective network management and secure routing between CH and CM. The clustering process is used to increase the throughput and decrease delay during data transmission. The proposed spectrum sensing and mobility management method increased the performance of spectral efficiency, detection probability, sensing delay, and detection accuracy. Secure routing is used to increase the throughput and packet delivery and reduce the routing overhead and route acquisition delay. Hybrid beamforming increased spectral efficiency, resulting in low SNR and computational efficiency. The overall process of the proposed work increases QoS compared to existing studies. The research highlights of this work are listed as follows:

- Spectrum scarcity and mobility are solved by performing secure spectrum sensing and handoff, which reduces the transmission delay and increases spectrum utilization. In addition, it mitigates SSDF and PUF attacks in the environment.
- To enhance hardware efficiency and spectral efficiency, we performed hybrid beamforming, and this provides secure hybrid beams, which increases signal quality and transmitting speed.
- To improve throughput and reduce network traffic during data transmission, we proposed secure routing using dingo optimization, which works against Sybil attacks and blackhole attacks.
- To efficiently manage the network, we performed the clustering and intersection of secure information provisioning, which helps prevent network collision and latency during vehicle communication.

## 6. Conclusions

The reliable and efficient framework, 6GCRN-IoCV, is proposed for effectively managing the spectrum mobility and network traffic. All entities such as PUs (ERSU), SUs, and pedestrians are authenticated to the blockchain to ensure authenticity by using the PUF, ID, and location. The authenticated nodes are allowed for cluster formation to manage the network by using an improved DBSCAN algorithm in consideration of metrics such as the direction, distance, local density, and location. CH is selected based on the maximum RSSI and trust. Intersection safety is ensured by constructing an intersection-aware map using the STLR algorithm. The spectrum scarcity issue is addressed by performing spectrum sensing in which Lite-CNN is exploited by considering the SNR, the trust factor for every time interval, and the noise level. The spectrum mobility is managed by effective spectrum handoff between SUs by considering metrics such as the noise, SNR, channel availability, congestion, and trust level. Spectrum mobility management reduces transmission delay. The communication among the vehicles is enhanced by performing optimal routing, which exploits the DOA algorithm by considering link stability, energy, trust, and relative velocity. The optimal next forwarder is selected by ERSU using the vehicle trajectory, the number of hops, vehicle speed, and link stability. Finally, the spectral efficiency is enhanced by hybrid beamforming by using multi-agent-based categorical DQN in which three agents are involved and actions taken by the agents are jointly assessed for effective hybrid beams. The proposed 6GCRN-IoCV method performs better than the existing works and obtains achievable results.

## Figures and Tables

**Table 1:** Notation table.

Notation	Description
$P_{d},T_{h},S_{e}$	Packet delivery ratio, throughput, spectral efficiency
U	Spectrum utilization
n	Local density
${℧}_{N}$	High local density SUs with a minimum density
$I_{m}$	Intersection aware map
$\;P_{i}\left(\sigma \right)$	Probability of arrival
$SC_{i}$	State of the channel
*d*	Distance between the prey and dingo
$\overset{´}{p}\;and\;p\;$	Position of the dingo
$m_{t},\;sT_{t},\;and\;re_{t}$	Action, state, and reward
$P^{⌀}$	Policy
$F^{RF}F^{BB}$	Analog precoder and digital precoder
$W^{RF}W^{BB}$	Analog combiner and digital combiner
$Q^{⌀}\left(sT_{t},m_{t}\right)$	Value function
Sc	Action space
A	Number of antennas

**Table 2:** Summary of related works.

References	Objectives	Methods or Algorithms Used	Limitations
[20]	Secure processing of data	BlockEd	Not suitable for the real-time scenario
[21]	Batch authentication	Batch authentication protocol and Byzantine fault tolerance consensus algorithm	Poor performance
[22]	Detection of a malicious user	SVM algorithm	Low scalability
[24]	Controlling of MRCSC	Cross-layer algorithm	Poor security
[25]	Spectrum access	MDP process	Inefficient spectrum sensing
[26]	Spectrum access and channel reservation	Hybrid dynamic channel reservation algorithm	Poor spectrum access
[27]	Spectrum handoff	DQN algorithm	High-security threats
[28]	Spectrum handoff	Trust factor	High transmission delay
[29]	Spectrum handoff	Probability prediction	Low Security
[30]	Task offloading	ILP and CHAT algorithm	Maximum energy consumption
[31]	Path selection and assigning channels	IBFD and OBFD methods	Inefficient path selection
[32]	Optimal routing	Social community partition algorithm	Less throughput
[33]	Delay aware routing	Binary tree replication algorithm and routing algorithm	Poor routing
[34]	Hybrid beamforming	Eigendecomposition method	Inefficient beam generation
[35]	Hybrid beamforming	IF baseband and active phased array	High hardware complexity

**Table 3:** Structure of Lite-CNN.

Layers		Size of Kernel	Filters	Stride
	Std. Conv.	1 × 5	5	1
	Max-Pool	1 × 2	5	2
Lite module	Squeeze Conv.	1 × 1	3	1
	Std. Conv	1 × 1	6	1
		1 × 2	6	1
		1 × 3	6	1
	Depth Conv.	1 × 2	6	1
		1 × 3	6	1
	Point Conv.	1 × 1	6	1
		1 × 1	6	1
	Max-Pool	1 × 2	18	2
			Dense	30
			Dense	20

**Table 4:** Simulation configurations.

Parameters	Values
Time taken for simulation	450 s
**Parameters for channel**	
Range of spectrum	10 to 500 MHz
Bandwidth of channel	30 MHz
No. of channels	30
Parameters for packets	
Interval of packets	2 s
Size of the packet	512
Generated packets numbers	1024
No. of packets	∼5000
**Parameters for 6G**	
Data rate	Maximum 2T bps
End-to-end delay	1 ms
Spectral efficiency	200 bps/Hz
**Parameters for network**	
Acceleration of vehicles	3.5 m/s${}^{2}$
No. of ERSUs	4
No. of vehicles	100
No. of secondary users	75
No. of primary users	5
Speed of the vehicle	10–25 m/s
Fusion centre numbers	2
Area of simulation	1250 × 1250

**Table 5:** Numerical analysis of the poposed vs. existing works.

Performance Metrics			NPH-CRN	MEC-ISS	CR-VANET	6GCRN IoCV
QoS Analysis	Throughput (kbps)	No of SUs	184	218	256	348
		No of Malicious SUs	128	146	166	200
	Packet Delivery Ratio (%)	No of SUs	69.6	74.4	79.6	89.6
		No of Malicious SUs	67.6	71.6	76.4	87.3
	Delay (ms)	No of SUs	110	100	90	70
		No of Malicious SUs	128.4	113	99	80.5
	Routing Overhead	No of SUs	90.4	80.5	72.3	63
	Route Acquisition Delay(ms)	No of routing hops	106.2	100.4	90	80.3
			**NPH-CRN**	**MEC-ISS**	**HBFMIMO**	**6GCRN IoCV**
Spectrum Efficiency Analysis	Probability of Detection	SNR	0.4	0.5	0.6	0.7
	Spectral Efficiency (%)	No of SUs	67.8	70.8	76	92.1
Spectrum Sensing Analysis	Total Transmit Power (dBm)	No of SUs	50	40.2	30.5	20
	Sensing Delay (ms)	Data Traffic Level	60	49.5	40.2	19.4

## Data Availability

Not applicable.
